# Can flexor tenosynovectomy and microsurgical epineurectomy improve clinical outcomes following open carpal tunnel release?

**DOI:** 10.1051/sicotj/2017009

**Published:** 2017-04-07

**Authors:** Serda Duman, Vedat Sahin, Hakan Sofu, Yalkin Camurcu, Hanifi Ucpunar

**Affiliations:** 1 Diyarbakir Selahaddin Eyyubi State Hospital 21100 Diyarbakir Turkey; 2 Baltalimani Bone and Joint Diseases Hospital 34470 Istanbul Turkey; 3 Faculty of Medicine, Erzincan University 24030 Erzincan Turkey; 4 Elbistan State Hospital 46300 Kahramanmaras Turkey

**Keywords:** Carpal tunnel, Median nerve, Flexor tenosynovectomy, Epineurectomy

## Abstract

*Introduction*: The purpose of this study was to comparatively evaluate the clinical outcomes of open carpal tunnel release with or without flexor tenosynovectomy and epineurectomy for the treatment of idiopathic carpal tunnel syndrome.

*Methods*: In this prospective single-blinded study, 61 wrists of 47 patients randomized to open carpal tunnel release without (Group-1) or with (Group-2) flexor tenosynovectomy and microsurgical epineurectomy. Physical examination including Phalen and Tinel’s signs, visible thenar atrophy, two-point discrimination, and grip strength measurement was performed. Visual Analogue Scale (VAS), Quick Disability of Arm Shoulder Hand (DASH) Questionnaire, Symptoms Severity Scale, Functional Status Scale, and electrophysiological study were assessed.

*Results*: The increase in the grip strength and Quick Disability of Arm Shoulder Hand Questionnaire score were significantly better in flexor tenosynovectomy and microsurgical epineurectomy group. The average pre-operative two-point discrimination was 6.3 ± 2 mm in Group-1 and 5.8 ± 1.7 mm in Group-2. Post-operatively at the end of 12 months, the mean two-point discrimination was measured as 5.9 ± 1.6 mm in Group-1 and 5.6 ± 1.3 mm in Group-2. When we compare the two groups according to the changes in VAS, Quick-DASH, symptoms severity scale, and functional status scale, only Quick-DASH score improvement was significantly better in Group-2 (*p* < 0.05). Improvements in VAS, symptoms severity scale, and functional status scale did not differ significantly.

*Conclusion*: We do not recommend routine flexor tenosynovectomy and microsurgical epineurectomy during open carpal tunnel release in patients with idiopathic carpal tunnel syndrome.

## Introduction

Carpal tunnel syndrome (CTS), which occurs due to symptomatic compression of the median nerve at the level of wrist joint, is the most common entrapment neuropathy with an estimated prevalence of 3.8% in the general population [1–4]. Although being a widely recognized syndrome in the clinical practice of medicine through decades since the first description by Paget in 1854, the exact etiology still remains unclear as the idiopathic CTS is the most common diagnosis [5]. The pathophysiology involves a combination of mechanical trauma, increased pressure, and ischemic injury to the median nerve within the carpal tunnel [6]. Several studies in the literature demonstrated that abnormalities of the synovial tissue inside the carpal tunnel may be related to the development of idiopathic CTS [7–9]. Thickening of the synovial tissue especially at the entrance and exit regions of the canal where the tendons slide over a fulcrum of the flexor retinaculum has been reported as leading to an increased pressure within the carpal tunnel [10]. Surgical treatment in the form of releasing the transverse carpal ligament either by open or endoscopic technique mainly aims to increase the space in the tunnel and hence reduce the interstitial pressure. Division of the transverse carpal ligament has been reported to yield excellent results in 75% of the patients [3]. As adjuncts in the surgical treatment, some authors perform flexor tenosynovectomy and/or microsurgical epineurotomy at the same session to obtain a more effective decompression [11, 12]. However, the positive or negative effects of these procedures on the clinical results of open surgical treatment of idiopathic CTS are controversial.

The purpose of this study was to comparatively evaluate the clinical outcomes of open carpal tunnel release with or without flexor tenosynovectomy and microsurgical epineurectomy for the treatment of idiopathic CTS.

## Materials and methods

In this prospective single-blinded study, 61 wrists of 47 patients who had undergone surgical treatment in Baltalimani Bone and Joint Diseases Education and Research Hospital between March 2009 and October 2012 were randomized to open surgical decompression with or without flexor tenosynovectomy and microsurgical epineurectomy after having approval from the local Ethical Research Committee. The exclusion criteria were CTS secondary to different etiologic factors, history of a rheumatologic or chronic inflammatory disease, thyroid anomalies, chronic renal failure, acromegaly, a medical history of corticosteroid injection to carpal tunnel and/or physical therapy and rehabilitation protocol for the wrist joint previously, a medical history of ipsilateral upper extremity fracture, and previous carpal tunnel surgery. The patients were randomized into two groups. Randomization was achieved via drawing lots with the help of an orthopedic surgery resident who did not know any of the patients and was completely blinded to their diagnosis, clinical features, and treatment method. Group-1 included 33 wrists of 25 patients treated by performing a standard open surgical release of transverse carpal ligament, whereas Group-2 included 28 wrists of 22 patients treated by performing an open surgical release of transverse carpal ligament combined with flexor tenosynovectomy and microsurgical epineurectomy at the same session. The flexor tenosynovectomy was not an extended removal of the entire synovial tissue surrounding all the nine flexor tendons running through the tunnel however, it consisted of the removal of the apparent hypertrophic synovial tissue around the tendons. On the other hand, microsurgical epineurectomy was performed as circumferential excision of the epineurium of the median nerve under the microscope. The tourniquet was routinely released prior to closure and bleeding control was also routinely performed; however, we did not encounter any clinical problem related to bleeding following flexor tenosynovectomy. In patients with bilateral CTS, both wrists underwent the same surgical technique in a sequential manner and thus they were included in the same study group. All patients were female with a mean age of 52.7 years (range, 39–75 years) in Group-1 and 52.5 years (range, 37–71 years) in Group-2. There was no specific reason or an intended choice for a gender distribution which did not include any male patients. Active and passive motion was permitted on the second post-operative day, standard wound care protocol was followed, and the sutures were removed by the fifteenth post-operative day in all cases. All wrists were followed up for a minimum of 12 months post-operatively.

A detailed physical examination included Phalen’s test, Tinel’s signs, two-point discrimination test, and an evaluation of thenar atrophy and grip strength, was performed in all patients pre- and post-operatively. The measurement of grip strength was carried out using a hydraulic grip dynamometer. Grip strength was first assessed pre-operatively and then at 12 months post-operatively. To perform the measurements, the patients sat with their arm raised in a position parallel to the body with the elbow flexed at 90°, and the forearm and wrist in neutral position. Three measurements with the maximum possible force were performed, and the highest values noted in kilograms-force. The Visual Analogue Scale (VAS) for pain, Quick Disability of Arm Shoulder Hand Questionnaire (Quick-DASH), Boston Questionnaire which is composed of the symptoms severity scale and functional status scale, and electrophysiological studies were used pre-operatively and at the end of 12 months post-operatively. All complications were noted.

Wilcoxon signed-rank test was applied to compare related data of pre-operative and post-operative periods, and nonparametric Mann-Whitney *U* test to compare independent interval data. The level of significance was set at *p* < 0.05.

## Results

The Phalen’s test was positive in 21 of 33 wrists in Group-1 and 20 of 28 wrists in Group-2 pre-operatively. The Tinel’s sign was positive in 17 wrists in Group-1 and 12 wrists in Group-2 pre-operatively. Visible thenar atrophy was detected in nine wrists in Group-1 and six wrists in Group-2. The average pre-operative two-point discrimination was 6.3 ± 2 mm in Group-1 and 5.8 ± 1.7 mm in Group-2. Post-operatively at the end of 12 months, the mean two-point discrimination was measured as 5.9 ± 1.6 mm in Group-1 and 5.6 ± 1.3 mm in Group-2. Table 1 summarizes the means, standard deviations, and minimum and maximum values of grip strength tests measured in pre- and post-operative periods. When we compare the forces measured in the pre-operative examination and 12 months after surgery, a 22% increase in grip strength was detected in Group-1 whereas it was 39% in Group-2 (*p* < 0.05).

**Table 1.** Grip strength measurements of Group-1 and Group-2.

		Pre-operative	12 months
Group-1	Mean	12.51	15.30
	Standard deviation	5.49	5.27
	Minimum	3	5
	Maximum	21	28
Group-2	Mean	13.46	18.75
	Standard deviation	6.03	6.82
	Minimum	5	5
	Maximum	24	32

In Group-1, the mean VAS pain score was 8.0 ± 1.06 points pre-operatively and it improved to 4.15 ± 1.0 points post-operatively (*p* < 0.05). The mean Quick-DASH score also improved from 71.6 ± 13.9 points to 50.9 ± 16.7 points (*p* < 0.05). The mean symptoms severity score was measured as 3.99 ± 0.7 points and functional status score as 2.85 ± 0.7 points pre-operatively whereas both were noted as 2.17 ± 0.9 points and 2.03 ± 0.9 points at 12 months after surgery (*p* < 0.05).

In Group-2, the mean VAS pain score was 8.0 ± 1.2 points pre-operatively and it improved to 3.39 ± 1.5 points post-operatively (*p* < 0.05). The mean Quick-DASH score also improved from 72.7 ± 14.6 points to 30.2 ± 23.1 points (*p* < 0.05). The mean symptoms severity score was measured as 3.94 ± 0.6 points and functional status score as 2.76 ± 0.7 points pre-operatively whereas both were noted as 2.12 ± 1.0 points and 1.65 ± 0.9 points at 12 months after surgery (*p* < 0.05).

**Figure 1.**
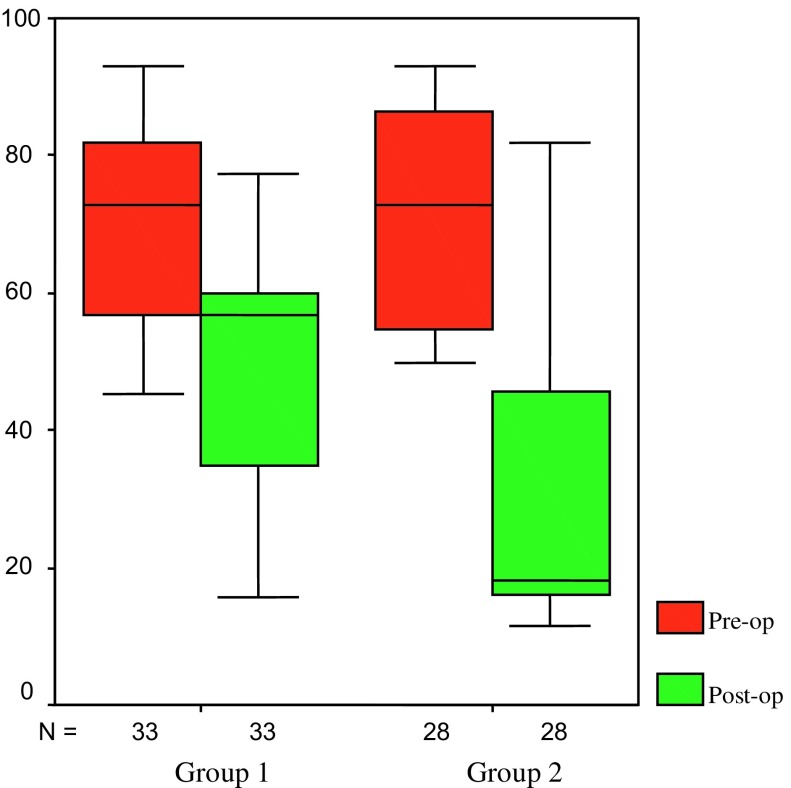
Graph demonstrating the statistical analysis of pre- and post-operative Quick-DASH scores.

When we compare the two groups according to the changes in VAS, Quick-DASH, symptoms severity scale, and functional status scale by applying Mann-Whitney *U* test, only Quick-DASH score improvement was significantly better in Group-2 (*p* < 0.05) (Figure 1). Improvements in VAS, symptoms severity scale, and functional status scale did not significantly differ according to whether or not tenosynovectomy and microsurgical epineurectomy were performed during open surgical release of the carpal tunnel.

Pre-operatively, the mean median distal motor latency was 4.60 ± 1.83 ms and the mean sensory nerve conduction velocity was 28.97 ± 17.63 m/s in Group-1 whereas they were 5.08 ± 2.46 ms and 25.80 ± 18.66 m/s in Group-2. Post-operatively, the mean distal motor latency was 4.02 ± 1.83 ms in Group-1 and 4.09 ± 1.07 ms in Group-2. The mean sensory nerve conduction velocity was 40.25 ± 11.81 m/s in Group-1 and 41.28 ± 10.57 m/s in Group-2 at 12 months after surgery. We could not detect any statistically significant difference between the two groups with respect to electrophysiological studies performed pre- and post-operatively (*p* > 0.05).

There was no wound infection in Group-1; whereas in Group-2, superficial wound infection was detected in one wrist and was treated successfully by oral antibiotics, debridement, and wound care. We did not detect any limitation in the range of motion of the operated wrists as well as the patients did not have difficulties with activities of daily living in both groups at 12 months after surgery. There were no differences between the groups with respect to presence of any pain or sensitivity on the scar tissue.

## Discussion

Theories regarding the pathophysiology behind CTS discussed in previous studies include repeated mechanical trauma, increased pressure inside the carpal tunnel, abnormalities of the synovial tissue, and chronic ischemic injury to the median nerve [6, 8, 13, 14]. Nakamichi and Tachibana reported that the transverse carpal ligament and tenosynovial tissue generally showed normal histology, and there were no typical or consistent changes with which idiopathic carpal tunnel syndrome could be associated [15]. On the other hand, in an experimental rabbit model, Lluch noted that similar tenosynovial changes were detected as found in tenosynovial specimens from patients who had had operative treatment of carpal tunnel syndrome [16]. Some investigators advocated performing flexor tenosynovectomy and/or microsurgical epineurectomy during carpal tunnel decompression to obtain a more effective decompression [11, 12, 17]. However, the benefit of these procedures for the surgical treatment of idiopathic carpal tunnel syndrome is also controversial. Our study was designed as a prospective randomized single-blinded trial and conducted to test whether flexor tenosynovectomy and microsurgical epineurectomy during open carpal tunnel decompression provide better clinical outcomes.

The sensitivity of Phalen’s test for diagnosing CTS has been reported between 67%–83%, and the sensitivity of Tinel’s sign has been reported between 48%–73% [18, 19]. Impaired two-point discrimination test and thenar muscle atrophy were also noted as signs of severe and prolonged median nerve compression [5, 20]. Measurement of grip strength is another useful method to evaluate hand function in patients with CTS [21]. Pre-operative clinical features of our patient groups in the aspects of the Phalen’s test, Tinel’s signs, visible thenar atrophy, two-point discrimination, and grip strength were similar. Post-operatively, the two-point discrimination improved without statistically significant difference between the two groups in our study, and the results were consistent with the results of previous studies [20]. Grip strength was found to be increased in both groups at 12 months after surgery. However, the increase in the grip strength was significantly better in wrists for which the flexor tenosynovectomy and microsurgical epineurectomy were added to standard open surgical release of the transverse carpal ligament. We agree with Ketchum who reported that a flexor tenosynovectomy would likely benefit workers who use the palm of the hand in heavy manual or highly repetitive work [12].

Although flexor tenosynovectomy and microsurgical internal neurolysis have been described as adjunct procedures which may be performed during open carpal tunnel decompression surgery, the positive or negative effects of these procedures on the clinical outcomes are controversial. In a study analyzing the cases re-operated for persistent symptoms following carpal tunnel surgery, fulminant synovitis was identified in all wrists [22]. Shum et al. mentioned that they observed neither an added benefit nor an increased rate of morbidity in association with the performance of a flexor tenosynovectomy at the time of carpal tunnel release [23]. Corradi et al. reported that significantly better results were obtained when a microsurgical internal neurolysis was added to the procedure, rather than simple decompression alone [11]. Borisch and Haussmann found no statistically significant difference between simple decompression and decompression combined with epineurotomy with regard to either the clinical or neurophysiological outcome [24]. Some authors noted that epineurotomy did not increase the rate of morbidity of carpal tunnel decompression but at the same time, did not result in improvement of the clinical outcomes with respect to standard surgical decompression without epineurectomy [25, 26]. On the other hand, Lundborg mentioned that intraneural scar tissue secondary to possible microvascular trauma during microsurgical internal neurolysis would create a risk of increasing post-operative morbidity [27]. In the present study only Quick-DASH score improvement was significantly better in wrists treated by performing an open surgical release of transverse carpal ligament combined with flexor tenosynovectomy and microsurgical epineurectomy. We could not detect any statistically significant difference between the two groups according to the evolution of the mean median distal motor latency and the mean sensory nerve conduction velocity measured by electrophysiological studies pre-operatively and 12 months after surgery.

Palmer and Toivonen concluded that, independent of the technique preferred open or endoscopic, their data supported the evidence that carpal tunnel release may not always be a safe and simple procedure [28]. We did not detect any major complication except one superficial wound infection which did not require secondary surgical intervention, and there was no significant increase in the complication rates related to the flexor tenosynovectomy and microsurgical epineurectomy.

In conclusion, we do not recommend routine flexor tenosynovectomy and microsurgical epineurectomy during open surgical carpal tunnel release in patients with idiopathic CTS. However, according to the results of our study, as we consider significant increase in the grip strength; flexor tenosynovectomy in the treatment of CTS would likely benefit the patients undertaking heavy manual work. Further studies are required to test the validity of our findings.

## Conflict of interest

There were not any conflicts of interest regarding the submission and publication of this manuscript.
